# Heteromeric Channels Formed From Alternating Kv7.4 and Kv7.5 α-Subunits Display Biophysical, Regulatory, and Pharmacological Characteristics of Smooth Muscle M-Currents

**DOI:** 10.3389/fphys.2020.00992

**Published:** 2020-08-12

**Authors:** Lyubov I. Brueggemann, Leanne L. Cribbs, Kenneth L. Byron

**Affiliations:** ^1^Department of Molecular Pharmacology and Neuroscience, Loyola University Chicago, Maywood, IL, United States; ^2^Department of Cell and Molecular Physiology, Loyola University Chicago, Maywood, IL, United States

**Keywords:** smooth muscle, Kv7.4, Kv7.5, α-subunit stoichiometry, M-current

## Abstract

Smooth muscle cells of the vasculature, viscera, and lungs generally express multiple α-subunits of the Kv7 voltage-gated potassium channel family, with increasing evidence that both Kv7.4 and Kv7.5 can conduct “M-currents” that are functionally important for the regulation of smooth muscle contractility. Although expression systems demonstrate that functional channels can form as homomeric tetramers of either Kv7.4 or Kv7.5 α-subunits, there is evidence that heteromeric channel complexes, containing some combination of Kv7.4 and Kv7.5 α-subunits, may represent the predominant configuration natively expressed in some arterial myocytes, such as rat mesenteric artery smooth muscle cells (MASMCs). Our previous work has suggested that Kv7.4/Kv7.5 heteromers can be distinguished from Kv7.4 or Kv7.5 homomers based on their biophysical, regulatory, and pharmacological characteristics, but it remains to be determined how Kv7.4 and Kv7.5 α-subunits combine to produce these distinct characteristics. In the present study, we constructed concatenated dimers or tetramers of Kv7.4 and Kv7.5 α-subunits and expressed them in a smooth muscle cell line to determine if a particular α-subunit configuration can exhibit the features previously reported for natively expressed Kv7 currents in MASMCs. Several unique characteristics of native smooth muscle M-currents were reproduced under conditions that constrain channel formation to a Kv7.4:Kv7.5 stoichiometry of 2:2, with alternating Kv7.4 and Kv7.5 α-subunits within a tetrameric structure. Although other subunit arrangements/combinations are not ruled out, the findings provide new insights into the oligomerization of α-subunits and the ways in which Kv7.4/Kv7.5 subunit assembly can affect smooth muscle signal transduction and pharmacological responses to Kv7 channel modulating drugs.

## Introduction

There are five pore-forming α-subunits (Kv7.1–Kv7.5) in the Kv7 family of voltage-gated potassium channels, each encoded by a single gene (*KCNQ1*–*KCNQ5*). In smooth muscle cells from various tissues, Kv7.4 and Kv7.5 have been implicated as the predominant contributors to native “M-currents,” the name given to the outwardly rectifying Kv7 currents in neurons, which were discovered based on their regulation by muscarinic acetylcholine receptor activation, and subsequently found to be regulated by multiple other G protein-coupled receptor types (Delmas and Brown, 2005). M-currents in smooth muscle cells have also demonstrated regulation by muscarinic acetylcholine receptor activation (Sims et al., 1985; Brueggemann et al., 2012), as well as other smooth muscle contractile and relaxant signal transduction pathways (Byron and Brueggemann, 2018b).

It is widely accepted that functional Kv7 channels form as tetramers of α-subunits. In cellular expression systems, overexpression of either Kv7.4 or Kv7.5 results in functional channels, presumably homomeric tetramers of Kv7.4 or Kv7.5 α-subunits, respectively. However, M-currents through these Kv7.4 and Kv7.5 homomeric channels have distinguishing features. More specifically, we found that Kv7.5 expressed alone yielded M-currents with a more negative V_0.5_ (−44 mV), compared with Kv7.4 expressed alone (−31 mV) (Brueggemann et al., 2011). In addition, Kv7.5 currents were strongly influenced by cellular signaling pathways—enhanced in amplitude by activation of cyclic adenosine monophosphate (cAMP)/protein kinase A (PKA) signaling, and inhibited by protein kinase C (PKC) signaling, whereas Kv7.4 currents were insensitive to these signaling pathways (Brueggemann et al., 2014b; Mani et al., 2016). Finally, a cyclooxygenase-2 inhibitor, diclofenac, induced a rapid and robust inhibition of Kv7.5 currents, but robustly enhanced the M-currents derived from expression of Kv7.4 alone (Brueggemann et al., 2011).

Exogenous *co-expression* of Kv7.4 with Kv7.5 could theoretically result in a mix of homomeric Kv7.4 and Kv7.5 channels, along with heteromeric tetramers that have a combination of both Kv7.4 and Kv7.5 α-subunits. Previous research, using a smooth muscle cell (A7r5 cell) expression system, revealed that M-currents in A7r5 cells co-expressing Kv7.4 with Kv7.5 had another distinct profile, with electrophysiological and pharmacological characteristics that could not be ascribed to a mix of Kv7.4 and Kv7.5 homomers (Brueggemann et al., 2011, 2014a,b; Mani et al., 2016). Co-expression of Kv7.4 and Kv7.5 yielded a voltage-dependent conductance that was well fit by a single Boltzmann function, with an intermediate V_0.5_ (−38 mV) (Brueggemann et al., 2011). The currents displayed partial responsiveness to cAMP/PKA or PKC-dependent regulation (Mani et al., 2016), and a slow moderate inhibition by diclofenac, likely reflecting M-currents generated predominantly by heteromeric Kv7.4/Kv7.5 channels. Notably, native M-currents in mesenteric artery smooth muscle cells (MASMCs), which express both Kv7.4 and Kv7.5, had electrophysiological and pharmacological characteristics that differed from those of Kv7.4 or Kv7.5 homomeric channels, more closely resembling the exogenously co-expressed Kv7.4/Kv7.5 profile.

We sought to determine what form of heteromeric Kv7.4/Kv7.5 tetramers would confer the M-current profile observed in cells that co-express Kv7.4 and Kv7.5. To more effectively control the α-subunit stoichiometry, we constructed concatenated dimers or tetramers of Kv7.4 and Kv7.5 α-subunits and expressed them in A7r5 cells. The resulting M-currents provide new insights into how the oligomerization of α-subunits can influence the biophysical and regulatory characteristics of heteromeric Kv7.4/Kv7.5 channels in smooth muscle cells.

## Materials and Methods

### Constructs

KCNQ5 cDNA encoding FLAG-tagged human Kv7.5 (accession number: AF202977), was a generous gift from Dr. T. Jentsch at the Max-Delbrück-Centrum for Molecular Medicine (Berlin, Germany), and human KCNQ4 (accession number: AF105202) was a generous gift from Dr. Ian Wood at the University of Leeds, Leeds, United Kingdom. Both cDNAs were subcloned into pIRES vectors (pIRES-FLAG-Q5, pIRES-Q4, respectively).

For construction of a Kv7.4-Kv7.5 (Q4–Q5) concatenated dimer vector, polymerase chain reaction (PCR) was used to link pIRES-Q4 to the 5′-end of FLAG-Q5, using primers to introduce *Bsm*BI-*Xho*I termini, targeting the 5′-end of FLAG-Q5 with pIRES-FLAG-Q5 as the template for PCR. The resulting product was inserted into the *Bsm*BI (nt 2040)-*Xho*I (vector) sites of pIRES-Q4. Finally, the *Bsr*GI (nt 343)-*Bsr*GI (vector) fragment of pIRES-FLAG-Q5 was inserted into the same sites of the partial construct resulting in pIRES-Q4-FLAG-Q5 (Q4–Q5).

To construct the reverse concatenated dimer, Kv7.5-Kv7.4 (Q5–Q4), the stop codon in the pIRES-FLAG-Q5 expression clone was eliminated by point mutation TAA to TAC. Polymerase chain reaction primers were used to amplify the first 270 nucleotides of Q4, adding an in-frame *Eco*RI site with the forward primer, using pIRES-Q4 as the template. The PCR product was joined to the 3′-end of pIRES-FLAG-Q5 at the in-frame *Eco*RI site, and lastly, the remaining portion of Q4 added using *Bsm*BI (nt 2014)-*Bam*HI (vector site).

A tetramer of four concatenated Kv7 subunits in the arrangement “Q5–Q4–Q5–Q4” was constructed by assembling the Kv7 dimers (each cloned into pIRES-2-EGFP) as follows. First, a silent point mutation introduced an *Eco*RV site in the Q5–Q4 dimer, creating “(Q5 + RV)–Q4.” An in-frame junction linker was created by PCR against the N-terminus of (Q5 + RV)–Q4, introducing 5′ *Bsm*BI and 3′ *Bam*HI, and the PCR product was cloned into Q5–Q4–*Bsm*BI-*Bam*HI (vector). Finally, the Q5–Q4–(Q5 + RV) partial construct was excised from the cloning vector using *Sna*BI (vector)-*Eco*RV, and inserted into the same sites of (Q5 + RV)–Q4 to create “Q5–Q4–Q5–Q4.”

QuikChange Lightning Site-Directed Mutagenesis Kit (Agilent Technologies) was used for point mutations. Polymerase chain reaction utilized Platinum II Hot Start PCR Master Mix (Invitrogen). All junctions were sequenced to confirm open reading frames, and all constructs are depicted schematically in Supplementary Figure S1.

### Cell Culture

A7r5 cells, an embryonic rat aortic smooth muscle cell line (Kimes and Brandt, 1976), were originally obtained from the ATCC (Byron and Taylor, 1993). A7r5 cells were cultured as described previously (Byron and Taylor, 1993). Kv7 constructs (described in section “Constructs”) were introduced into subcultured A7r5 cells by transient transfection with Lipofectamine 3000^®^ transfection reagent according to the manufacturer’s protocol. A7r5 cells expressing the Kv7 constructs described in section “Constructs” were used for electrophysiological studies 7–14 days after transfection. No antibiotic resistance selection marker was utilized, but rather cells were selected based on the detection of EGFP fluorescence (EGFP was co-expressed via the bi-cistronic expression vector). The relatively long time (7–14 days) after transfection is intended to address the potential mismatch between overexpressed channels and natively expressed receptor signaling pathways. Previous studies have suggested that robust currents through exogenously expressed Kv7.5 channels in A7r5 cells are initially relatively insensitive to regulation by activation of natively expressed vasopressin receptors, but the signaling capacity adjusts with time after transfection, stabilizing in a week to 10 days to produce regulatory responses similar to those observed with natively expressed Kv7.5 (Byron and Brueggemann, 2018a).

### Electrophysiology

We recorded whole cell M-currents using a perforated patch configuration under voltage-clamp conditions. Note that M-currents through exogenously expressed Kv7 channels are approximately 100-fold greater in amplitude than the M-currents recorded through natively expressed Kv7.5 in A7r5 cells (Brueggemann et al., 2009, 2011); the contributions of native Kv7.5 currents to the recordings were therefore deemed negligible. All experiments were conducted at room temperature (20°C) with continuous perfusion of bath solution as described previously (Brueggemann et al., 2007). The standard bath solution contained (in mM): 5 KCl, 130 NaCl, 10 HEPES, 2 CaCl_2_, 1.2 MgCl_2_, 5 glucose, pH 7.3. Standard internal (pipette) solution contained (in mM): 110 K gluconate, 30 KCl, 5 HEPES, 1 K_2_EGTA, pH 7.2. Osmolality was adjusted to 270 mOsm/l with D-glucose. Amphotericin B (120 μg/ml) in the internal solution was used for membrane patch perforation. An Axopatch 200B amplifier, under control of PCLAMP10 software, was used to generate voltage-clamp command voltages. Series resistances after amphotericin perforation were 8–15 MΩ and were compensated by 60%. Whole-cell currents were digitized at 2 kHz and filtered at 1 kHz, respectively. Prior to treatments, stable M-currents were recorded under control conditions for at least 15 min. M-currents were recorded using a 5 s voltage step protocol from a −74 mV holding potential to test potentials ranging from −124 to −6 mV, followed by a 1 s step to −120 mV (values were corrected for liquid junction potential offline). The instantaneous tail current amplitude (estimated from exponential fit of current deactivation measured at −120 mV) was converted to conductance according to the equation: *G* = *I*_tail_/(−120 - *E*_K_), where *I*_tail_ is the instantaneous tail current amplitude, −120 mV is the tail current step potential and *E*_K_ is the reversal potential for potassium (−86 mV). The resulting conductance plots, recorded in untreated (control) or treated cells, were fitted to a Boltzmann distribution: *G(V)* = *G*_max_/[1 + exp(*V*_0.5_ - *V*)/*k*], where *G* is conductance, *G*_max_ is a maximal conductance, *V*_0.5_ is the voltage of half-maximal activation and *k* is the slope factor.

### Statistical Analysis

Data were analyzed using SigmaPlot (Systat Software, Inc., San Jose, CA, United States). Data are expressed as mean ± standard error of the mean (SEM). Sample size for each group was not pre-determined, but instead was based on effect size and intrinsic variability within each group under the recording and treatment conditions that were applied. Because of variations in expression levels and cell sizes, current amplitudes for individual cell I–V curves were normalized to the current amplitude achieved with a voltage step to −6 mV under control conditions; all statistical comparisons were performed on normalized data. Two-tailed paired Student’s *t*-tests were used to compare parameters measured before and after treatments. For comparisons between two independent groups, two-tailed *t*-tests were used. Differences among multiple treatment groups were assessed by analysis of variance (ANOVA) followed by a Holm−Sidak *post hoc* test. Differences were considered statistically significant with *P* values ≤ 0.05. All groups were included in multiple groups ANOVA testing with no exceptions and results from all performed intergroup comparisons are reported irrespective of outcome.

## Results

### Kv7.4–Kv7.5 (Q4–Q5) Dimers

**FIGURE 1:**
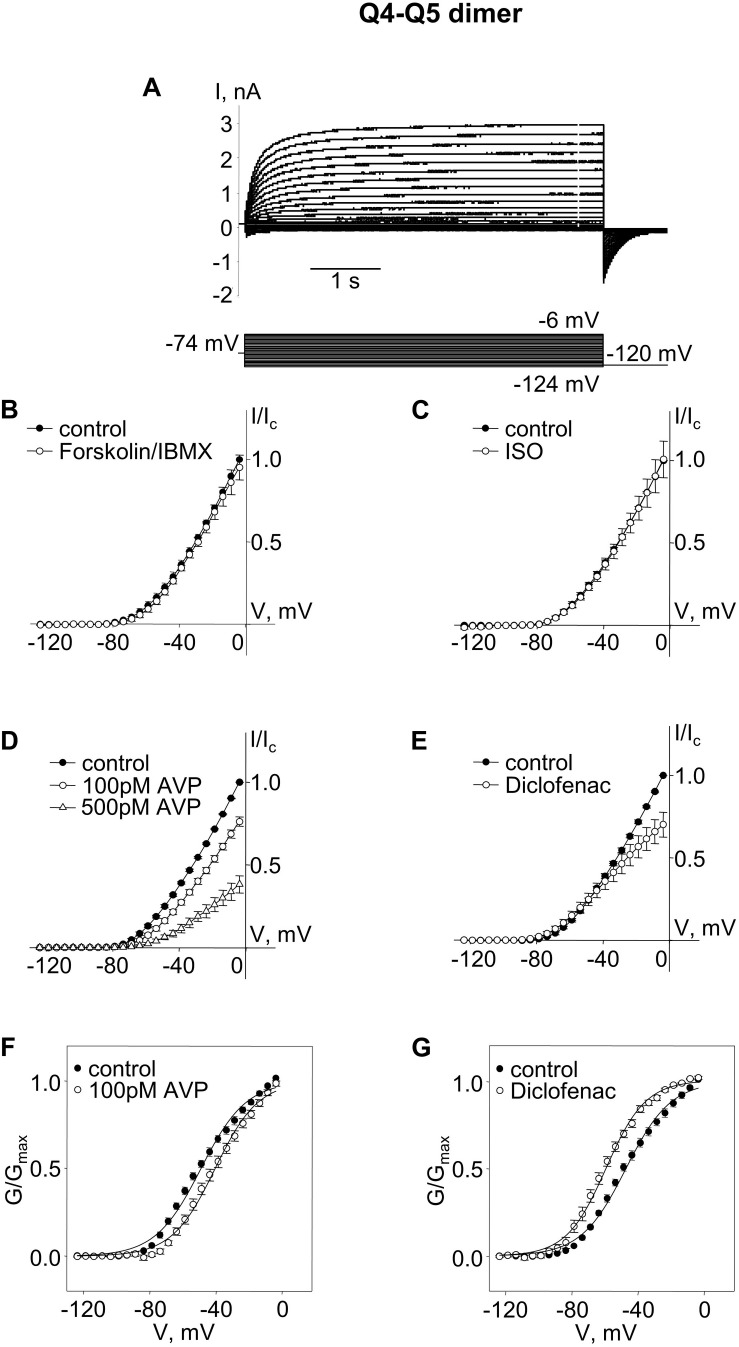
M-currents through channels formed from Kv7.4–7.5 (Q4–Q5) dimers were insensitive to cAMP/PKA activation but were sensitive to AVP and diclofenac. **(A)** Representative superimposed raw current traces recorded from a cell expressing Q4–Q5 dimers subjected to a series of voltage steps applied from a holding voltage of −74 mV to test potentials ranging from −124 to −6 mV, followed by a step back to −120 mV. **(B–E)** Average normalized current–voltage (I–V) relationships in A7r5 cells expressing Q4–Q5 dimers, before (closed circles) and during application of **(B)** 10 μM forskolin in combination with 500 μM IBMX (Forskolin/IBMX for 15 min, open circles, *n* = 4), **(C)** 1 μM isoproterenol (ISO for 15 min, open circles, *n* = 4), **(D)** AVP (100 pM AVP for 15 min, open circles, followed by 500 pM AVP for 15 min open triangles, *n* = 4), **(E)** diclofenac [100 μM for 15 min, open circles, *n* = 4, **(D)**]. **(F,G)** Mean fractional conductance plots calculated from tail currents measured before (closed circles) and during application of **(F)** 100 pM AVP (open circles, *n* = 4) or **(G)** 100 μM diclofenac (open circles, *n* = 4) fitted to the Boltzmann distribution.

To intentionally generate M-currents specifically from Kv7.4/Kv7.5 heteromers, we constructed an expression vector (Q4–Q5) designed to produce a concatenated dimer, with the N-terminus of Kv7.5 tethered to the C-terminus of Kv7.4, with the expectation that the expressed dimer pairs would assemble to form tetrameric channels containing two Kv7.4 α-subunits and two Kv7.5 α-subunits. Expression of this construct in A7r5 cells resulted in M-currents similar to those previously recorded when Kv7.4 and Kv7.5 were co-expressed via separate vectors in A7r5 cells, with an outwardly rectifying current-voltage profile, but with a significantly more negative V_0.5_ of approximately −48 mV [Figure 1; V_0.5_ was ∼−40 mV when Kv7.4 and Kv7.5 were co-expressed (Brueggemann et al., 2011, 2014b)].

We then tested treatments previously found to distinguish among Kv7.4 homomers, Kv7.5 homomers, and Kv7.4/Kv7.5 heteromers. Neither a robust cAMP/PKA-activating stimulus [combination of the adenylyl cyclase activator, forskolin (10 μM) with a cAMP phosphodiesterase inhibitor, isobutylmethylxanthine (IBMX, 500 μM)], nor a more physiological cAMP/PKA-activating stimulus (the β-adrenergic receptor agonist, isoproterenol, 1 μM), had any significant effect on M-currents via channels formed from Q4–Q5 dimers (Figures 1B,C; effects of treatments on current amplitude were assessed based on differences between currents measured following a voltage step to −20 mV under control conditions (before treatment) and following treatment for 10 min, using a paired Student’s *t*-test). In contrast, treatment with arginine–vasopressin (AVP, 100 pM, 500 pM, a PKC-activating stimulus) induced a significant concentration-dependent suppression of the current amplitude (∼25% decrease, on average, at −20 mV with 100 pM AVP, *P* = 0.005 based on a paired Student’s *t*-test, *n* = 4). The suppression of the current amplitude by 100 pM AVP was associated with a significant positive shift of the conductance plot (∼9 mV on average, *P* = 0.007, *n* = 4, Figures 1D,F; effects of treatments on voltage-dependence of activation were assessed based on differences between V_0.5_ measured under control conditions (before treatment) and following treatment for 10 min, using a paired Student’s *t*-test). Diclofenac (100 μM), was previously found to block Kv7.5 homomers, but to robustly enhance Kv7.4 homomers. The same treatment induced a very modest suppression of Q4–Q5 currents at voltages between −40 and 0 mV (Figure 1E). This effect of 100 μM diclofenac was associated with an approximately 12 mV negative shift of the Q4–Q5 voltage dependence of activation (a significant change in V_0.5_, based on a paired Student’s *t*-test, *P* = 0.022, *n* = 4, Figure 1G), similar to its previously reported effect on M-currents in A7r5 cells that co-expressed Kv7.4 and Kv7.5 (Brueggemann et al., 2011).

### Kv7.5–Kv7.4 (Q5–Q4) Dimers

A cAMP/PKA-dependent enhancement of currents through Kv7.5 channels (and to a lesser extent though heteromeric Kv7.4/Kv7.5 channels) was previously attributed to phosphorylation of a serine residue (S53) found on the cytosolic N-terminal segment of the Kv7.5 α-subunit (but absent on Kv7.4, which, when expressed alone, was insensitive to cAMP/PKA-activating stimuli) (Brueggemann et al., 2018, 2020). The results shown in Figures 1B,C suggest that, unlike previous recordings of M-currents through co-expressed Kv7.4 and Kv7.5, M-currents through channels formed from Q4–Q5 dimers were completely insensitive to cAMP/PKA-activating stimuli. We considered that perhaps the tethering of the Kv7.5 N-terminus to the C-terminus of Kv7.4 in channels formed by the Q4–Q5 dimers might constrain the position of the S53 site and hence render the channels insensitive or unresponsive to regulation by PKA.

**FIGURE 2:**
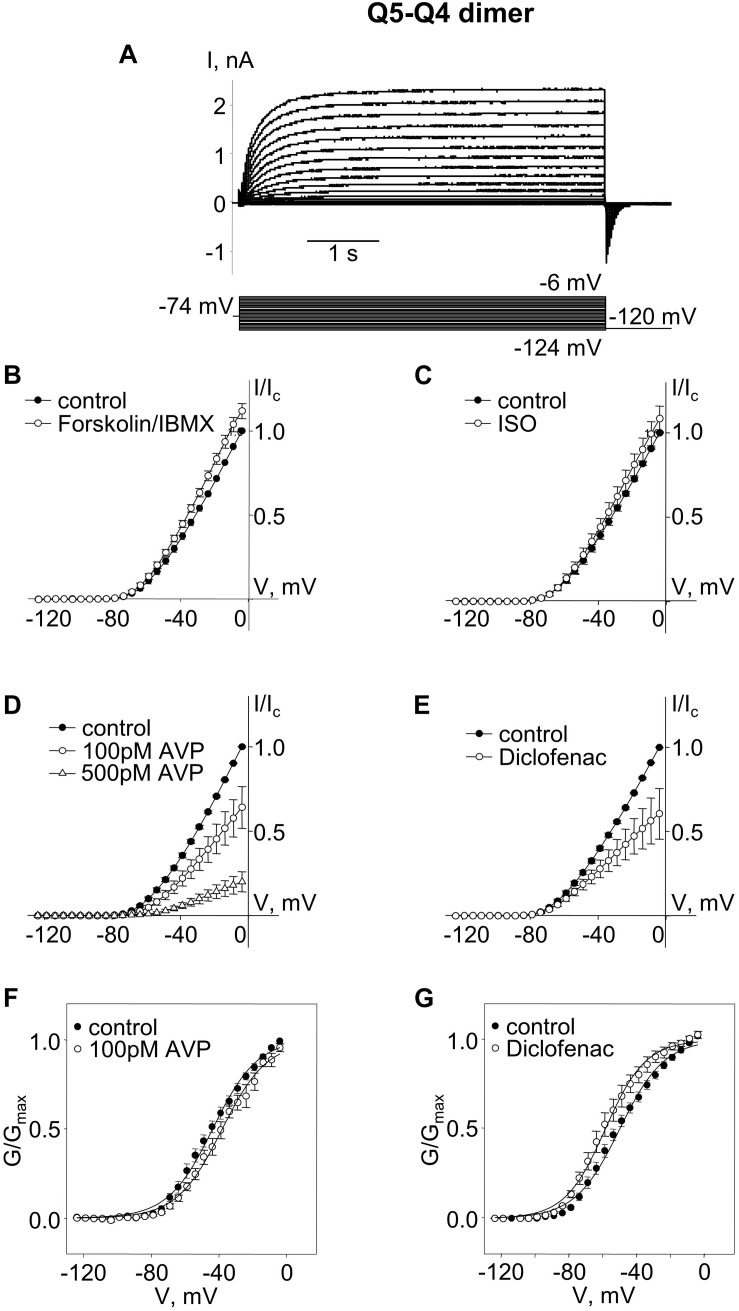
M-currents through channels formed from Kv7.5–7.4 (Q5–Q4) dimers were sensitive to cAMP/PKA activation and sensitive to AVP and diclofenac. **(A)** Representative superimposed raw current traces recorded from a cell expressing Q5–Q4 dimers subjected to a series of voltage steps applied from a holding voltage of −74 mV to test potentials ranging from −124 to −6 mV, followed by a step back to −120 mV. **(B–E)** Average normalized current–voltage (I–V) relationships of Q5–Q4 dimer before (closed circles) and during application of 10 μM forskolin in combination with 500 μM IBMX [Forskolin/IBMX for 15 min, open circles, n = 6, **(B)**], 1 μM isoproterenol [ISO for 15 min, open circles, n = 4, **(C)**], AVP [100 pM AVP for 15 min, open circles, followed by 500 pM AVP for 15 min open triangles, *n* = 5, **(D)**], diclofenac [100 μM for 15 min, open circles, *n* = 4, **(E)**]. **(F,G)** Mean fractional conductance plots calculated from tail currents measured before (closed circles) and during application of 100 pM AVP [open circles, *n* = 5, **(F)**] or 100 μM diclofenac [open circles, *n* = 4, **(G)**] fitted to the Boltzmann distribution.

We constructed an expression vector for concatenated Q5–Q4 dimers, with α-subunits assembled in the opposite order to free up the Kv7.5 N-terminus S53 site (in this case the C-terminus of Kv7.5 was tethered to the N-terminus of Kv7.4). Expression of the Q5–Q4 dimer construct yielded M-currents with a V_0.5_ of ∼−47 mV, similar to Q4–Q5, but in this case the currents were modestly, but significantly, enhanced by forskolin/IBMX treatment, and tended to be slightly enhanced in the presence of isoproterenol, though that effect was not significant. The effects of AVP and diclofenac were similar to what was observed for Q4–Q5 dimers, both in terms of suppression of current amplitude and the opposite shifts in voltage dependence (Figure 2).

### Kv7.5–Kv7.4–Kv7.5–Kv7.4 (Q5–Q4–Q5–Q4) Tetramers

**FIGURE 3:**
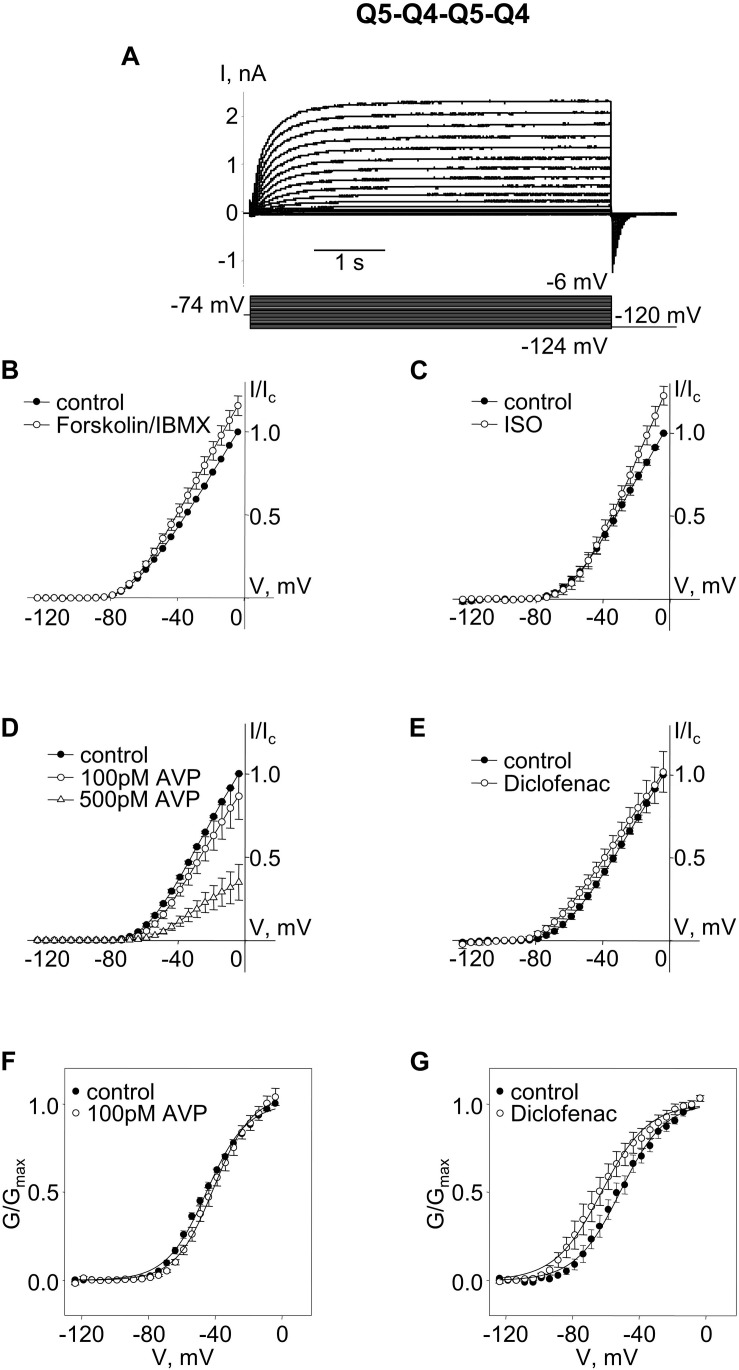
M-currents through channels formed from concatenated Kv7.5–7.4–7.5–7.4 (Q5–Q4–Q5–Q4) tetramers were sensitive to cAMP/PKA activation and diclofenac, and sensitive to 500 pM AVP. **(A)** Representative superimposed raw current traces recorded from a cell expressing Q5–Q4–Q5–Q4 tetramers subjected to a series of voltage steps applied from a holding voltage of −74 mV to test potentials ranging from −124 to −6 mV, followed by a step back to −120 mV. **(B–E)** Average normalized current–voltage (I–V) relationships of concatenated Q5–Q4–Q5–Q4 before (closed circles) and during application of 10 μM forskolin in combination with 500 μM IBMX [Forskolin/IBMX for 15 min, open circles, *n* = 6, **(B)**], 1 μM isoproterenol [ISO for 15 min, open circles, *n* = 4, **(C)**], AVP [100 pM AVP for 15 min, open circles, followed by 500 pM AVP for 15 min open triangles, *n* = 5, **(D)**], diclofenac [100 μM for 15 min, open circles, *n* = 6, **(E)**]. **(F,G)** Mean fractional conductance plots calculated from tail currents measured before (closed circles) and during application of 100 pM AVP [open circles, *n* = 5, **(F)**] or 100 μM diclofenac [open circles, *n* = 6, **(G)**] fitted to the Boltzmann distribution.

The Q5–Q4 dimer expression yielded M-currents with several characteristics that matched previous measurements of M-current in A7r5 cells co-expressing Kv7.4 and Kv7.5, supporting a likely tetramer stoichiometry of two Kv7.4 and two Kv7.5. Although the dimers might be expected to assemble in a head-to-tail format with alternating α-subunits (e.g., Q4–Q5–Q4–Q5), we could not rule out a side-by-side assembly, such that like α-subunits are adjacent (e.g., Q4–Q4–Q5–Q5). To force an alternating α-subunit assembly, we made an expression vector for a tetrameric concatemer of Q5–Q4–Q5–Q4. Expression of this Q5–Q4–Q5–Q4 construct in A7r5 cells resulted in M-currents (Figure 3A) that resembled those from the Q5–Q4 dimers in terms of V_0.5_ (∼−51 mV) and responses to forskolin/IBMX or isoproterenol (Figures 3B,C). These M-currents were slightly less sensitive to AVP, with an insignificant suppression of current amplitude or shift in V_0.5_ by 100 pM AVP, but a significant suppression of current amplitude by 500 pM AVP (Figures 3D,F). Diclofenac did not significantly alter the current amplitude, but there was still a ∼12 mV mean negative shift in the *V*_0.5_ of Q5–Q4–Q5–Q4 M-currents in the presence of diclofenac [Figures 3E,G, *p* = 0.022 (Student’s paired *t*-test), *n* = 6].

### M-Current Comparisons Among Kv7.4/Kv7.5 Heteromers From Co-expression, Dimer Expression, or Tetramer Expression

#### cAMP/PKA-Activating Stimuli

**FIGURE 4:**
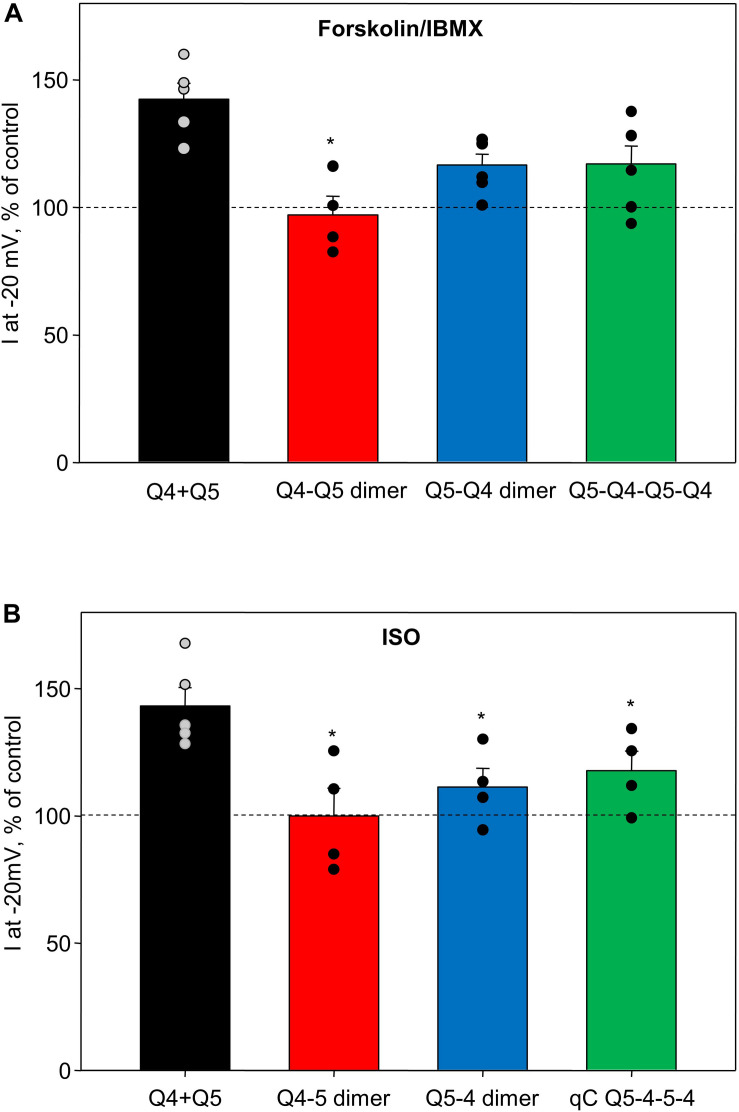
Summary of forskolin/IBMX and isoproterenol-induced increase in current amplitude of Q4–Q5 dimers, Q5–Q4 dimers, and Q5–Q4–Q5–Q4 tetramers, in comparison with M-currents through channels formed from co-expressed individual Kv7.4 and Kv7.5 α-subunits (Q4 + Q5). **(A)** Summarized bar graph of forskolin/IBMX (10 and 500 μM, respectively) -induced current enhancement through co-expressed individual Kv7.4 and Kv7.5 channels (Q4 + Q5, black, *n* = 5), Q4–Q5 dimer (red, *n* = 4), Q5–Q4 dimers (blue, *n* = 6), and Q5–Q4–Q5–Q4 tetramers (green, *n* = 6), measured at −20 mV. *Significant difference from co-expressed individual Kv7.4 and Kv7.5 channels (*P* = 0.002, One Way ANOVA). **(B)** Summarized bar graph of isoproterenol (1 μM)-induced current enhancement through co-expressed individual Kv7.4 and Kv7.5 channels (Q4 + Q5, black, *n* = 5), Q4–Q5 dimer (red, *n* = 4), Q5–Q4 dimers (blue, *n* = 4), and Q5–Q4–Q5–Q4 tetramer (green, *n* = 4), measured at −20 mV. *Significant difference from co-expressed individual Kv7.4 and Kv7.5 channels (*P* = 0.015, One Way ANOVA). Dashed lines indicate control current level.

We compared the enhancing effects of forskolin/IBMX (Figure 4A) or isoproterenol (Figure 4B) on M-currents in A7r5 cells expressing Q4–Q5 dimers, Q5–Q4 dimers, or Q5–Q4–Q5–Q4 tetramers to M-currents measured in A7r5 cells co-expressing Kv7.4 and Kv7.5 (Q4 + Q5, based on data from results originally reported in Mani et al., 2016). Channels that formed from Q4–Q5 dimers were unresponsive to either the more robust cAMP/PKA-activating stimulus (forskolin/IBMX), or to isoproterenol (in both cases, significantly less responsive than channels formed from co-expressed Kv7.4 and Kv7.5). Expression of the reverse dimer (Q5–Q4) and the Q5–Q4–Q5–Q4 constructs produced M-currents that responded to forskolin/IBMX like the co-expressed Kv7.4/Kv7.5 M-currents, but they were slightly less sensitive to isoproterenol.

#### AVP and Diclofenac Effects on M-Current Amplitude

**FIGURE 5:**
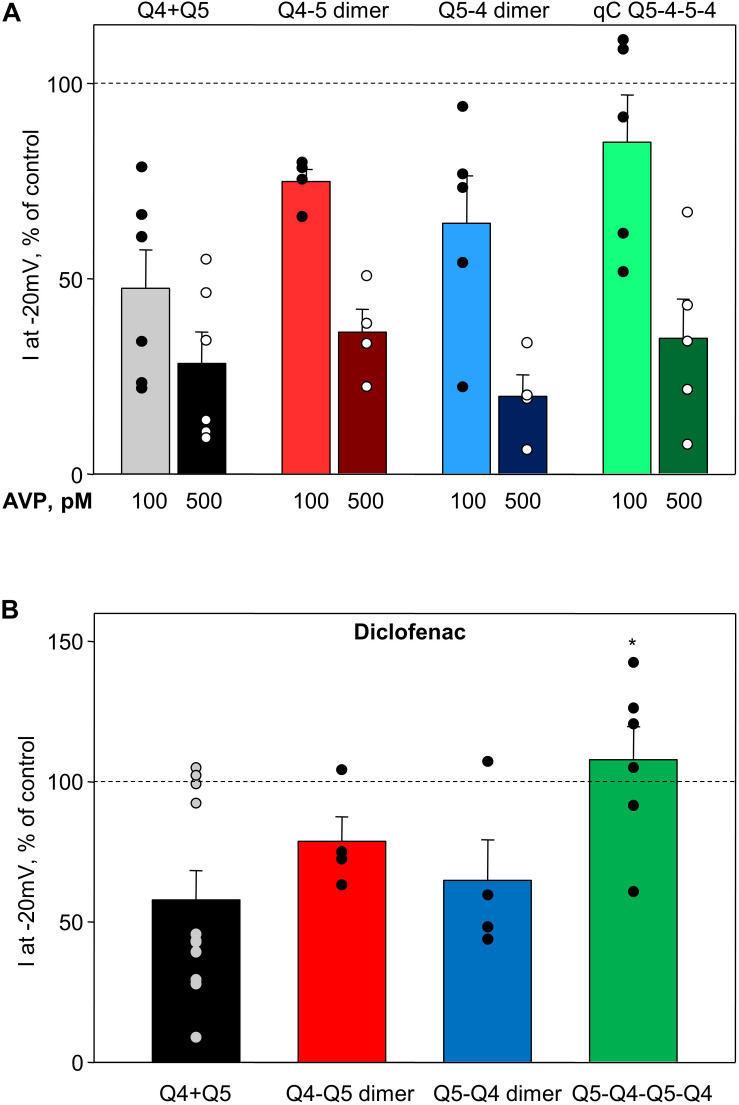
Summary of AVP and diclofenac-induced decrease in M-current amplitude of Q4–Q5 dimers, Q5–Q4 dimers, and Q5–Q4–Q5–Q4 tetramers, in comparison with M-currents through channels formed from co-expressed individual Kv7.4 and Kv7.5 α-subunits (Q4 + Q5). **(A)** Summarized bar graph of AVP (100 pM light color and 500 μM dark color, respectively)-induced current suppression through co-expressed individual Kv7.4 and Kv7.5 α-subunits (Q4 + Q5, gray/black, *n* = 6), Q4–Q5 dimers (red, *n* = 4), Q5–Q4 dimers (blue, *n* = 5), and Q5–Q4–Q5–Q4 tetramers (green, *n* = 5), measured at −20 mV. **(B)** Summarized bar graph of diclofenac (100 μM)-induced current suppression through co-expressed individual Kv7.4 and Kv7.5 α-subunits (Q4 + Q5, black, *n* = 11), Q4–Q5 dimer (red, *n* = 4), Q5–Q4 dimers (blue, *n* = 4), and Q5–Q4–Q5–Q4 tetramers (green, *n* = 6), measured at −20 mV. *Significant difference from co-expressed individual Kv7.4 and Kv7.5 channels (*P* = 0.031, One Way ANOVA). Dashed lines indicate control current level.

Arginine–vasopressin induced a similar concentration-dependent suppression of current amplitude in heteromeric Kv7.4/Kv7.5 channels regardless of whether they formed by co-expression of the individual α-subunits or by expression of Q4–Q5 dimers, Q5–Q4 dimers, or Q5–Q4–Q5–Q4 tetramers (Figure 5A). Diclofenac reduced M-current amplitude at −20 mV in cells co-expressing Kv7.4 and Kv7.5, though there was considerable variability in the extent of the effect, with some cells unaffected and others with robust suppression of the M-currents (Figure 5B, Q4 + Q5, black bar, based on results from Brueggemann et al., 2011). Cells expressing Q4–Q5 or Q5–Q4 dimers displayed a somewhat more uniform modest suppression of M-currents by diclofenac (Figure 5B, red and blue bars, respectively), with a mean response that did not differ significantly from the Kv7.4 and Kv7.5 co-expressing cells (Q4 + Q5). The tetrameric Q5–Q4–Q5–Q4 construct was significantly less affected by diclofenac in terms of its M-current amplitude at −20 mV (Figure 5B, green bar).

#### Voltage-Dependence of Activation

**FIGURE 6:**
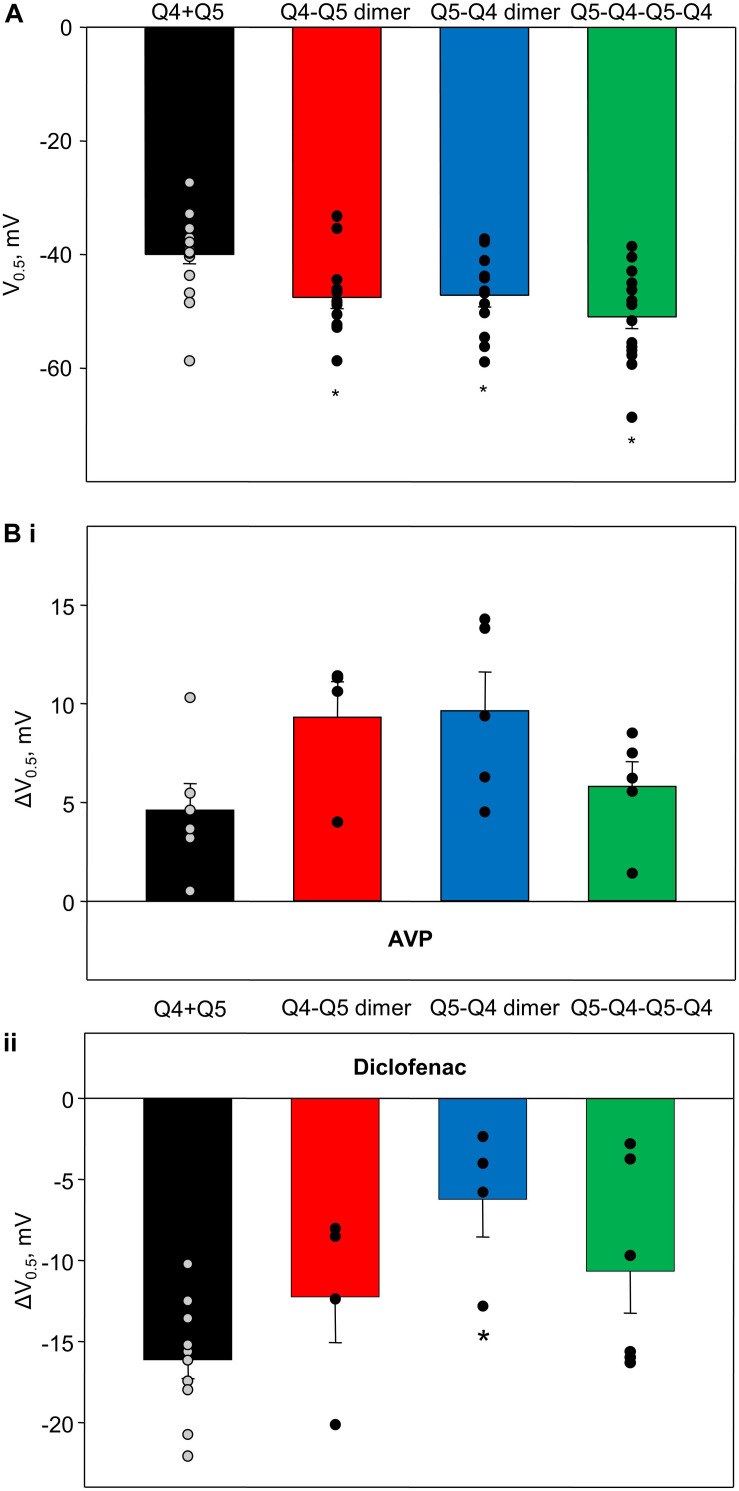
Voltage of half-maximal activation of M-currents through channels formed from Q4–Q5 dimers, Q5–Q4 dimers, and Q5–Q4–Q5–Q4 tetramers, in comparison with co-expressed individual Kv7.4 and Kv7.5 α-subunits (Q4 + Q5), and effects of AVP and diclofenac on voltage of half-maximal activation of corresponding channels. **(A)** Voltage of half-maximal activation (V_0.5_) of Kv7.4/Kv7.5 channels formed by co-expressed individual Kv7.4 and Kv7.5 V (Q4 + Q5, black, *n* = 17), Q4–Q5 dimers (red, *n* = 12), Q5–Q4 dimers (blue, *n* = 13), and Q5–Q4–Q5–Q4 tetramers (green, *n* = 17). *Significant difference from co-expressed individual Kv7.4 and Kv7.5 channel α-subunits (*P* < 0.001, One Way ANOVA). **(Bi)** AVP (100 pM)-induced positive shift of V_0.5_ of Kv7.4/Kv7.5 channels formed by co-expressed individual Kv7.4 and Kv7.5 α-subunits (Q4 + Q5, black, *n* = 6), Q4–Q5 dimers (red, *n* = 4), Q5–Q4 dimers (blue, *n* = 5), and Q5–Q4–Q5–Q4 tetramers (green, *n* = 5). **(Bii)** Diclofenac (100 μM)-induced negative shift of V_0.5_ of Kv7.4/Kv7.5 channels formed by co-expressed individual Kv7.4 and Kv7.5 α-subunits (Q4 + Q5, black, *n* = 11), Q4–Q5 dimers (red, *n* = 4), Q5–Q4 dimers (blue, *n* = 4), and Q5–Q4–Q5–Q4 tetramers (green, *n* = 6). *Significant difference from co-expressed individual Kv7.4 and Kv7.5 α-subunits (*P* = 0.016, One Way ANOVA).

The voltages of half-maximal activation (V_0.5_) of Kv7.4/Kv7.5 heteromers formed by co-expressed individual Kv7.4 and Kv7.5 α-subunits (Q4 + Q5, based on results from Brueggemann et al., 2011, 2014b; Mani et al., 2016), Q4–Q5 dimers, Q5–Q4 dimers, and Q5–Q4–Q5–Q4 tetramers are compared in Figure 6A. The dimeric and tetrameric constructs all formed channels that activate at significantly more negative voltages (Figure 6A, red, blue, and green bars), compared with channels formed as a result of co-expression of the individual α-subunits (Figure 6A, black bar). However, the effects of 100 pM AVP and diclofenac on V_0.5_ were not significantly different among channels formed from co-expressed or concatenated constructs: AVP induced a positive shift of V_0.5_, ranging from ∼4 to 9 mV (Figure 6Bi), while diclofenac (100 μM) induced a negative shift that tended to be greater in co-expressed Q4 + Q5 channels (Figure 6Bii, black bar), but was not significantly different from the effect measured in cells expressing the dimeric or tetrameric constructs (Figure 6Bii, red, blue, and green bars).

## Discussion

Heteromeric channels containing Kv7.4 and Kv7.5 α-subunits remain among the least characterized subtypes of Kv7 channels despite increasing evidence that they are likely the predominant contributors to M-currents in many types of smooth muscle cells. A fundamental unanswered question is how do the α-subunits assemble to account for the properties of M-currents recorded in smooth muscle myocytes? Expressing concatenated dimers of Kv7.4 and Kv7.5, and concatenated tetramers with alternating Kv7.5 and Kv7.4 α-subunits in a smooth muscle cell line revealed that the previously reported unique characteristics of native smooth muscle M-currents can be largely reproduced under conditions that favor a 2 Kv7.4: 2 Kv7.5 stoichiometry, with alternating Kv7.4 and Kv7.5 α-subunits within a tetrameric structure. The results further revealed that responses to cAMP/PKA-activating stimuli appear to require unconstrained Kv7.5 N-termini for full effect.

### Heteromeric Kv7.4/Kv7.5 Channels Behave Differently From Homomeric Kv7.4 and Homomeric Kv7.5 in a Smooth Muscle Expression System

Previous studies had revealed some notable differences in the effects of treatments on homomeric Kv7.4 and homomeric Kv7.5 currents using A7r5 smooth muscle cells as an expression system. Mani et al. reported that the combination of forskolin (10 μM) with IBMX (500 μM) increased current amplitude at −20 mV by ∼2.5-fold in cells overexpressing Kv7.5 channels, but had no detectable effect when Kv7.4 was overexpressed by itself (Mani et al., 2016). In another previous study, in A7r5 cells overexpressing Kv7.5 alone, AVP treatment significantly reduced Kv7.5 current amplitude at −20 mV by 66 ± 7% (*n* = 9) and 80 ± 6% (*n* = 6), with 100 and 500 pM AVP, respectively; in contrast, in A7r5 cells overexpressing Kv7.4 alone, AVP (100 or 500 pM) had no significant effect on the current amplitude (Brueggemann et al., 2014b). We also previously found that a pharmacological agent, diclofenac, had diametrically opposite effects on currents via Kv7.4 and Kv7.5 homomers overexpressed in A7r5 cells (Brueggemann et al., 2011). Stable Kv7.5 currents recorded at a −20 mV holding voltage were abruptly inhibited by 100 μM diclofenac treatment, whereas the same treatment in A7r5 cells overexpressing Kv7.4 alone resulted in ∼1.5-fold increase in current amplitude at the same holding voltage (Brueggemann et al., 2011).

In each of the previous studies (Brueggemann et al., 2011, 2014b; Mani et al., 2016), the effects of the treatments on cells co-expressing Kv7.4 and Kv7.5 were distinct from those elicited when either subunit was expressed alone. Notably the responses of the cells and the characteristics of the currents measured in A7r5 cells co-expressing Kv7.4 and Kv7.5 were similar to those measured in freshly isolated arterial myocytes from rat mesenteric arteries (Mackie et al., 2008; Brueggemann et al., 2011; Mani et al., 2016). Proximity ligation assays and results of co-immunoprecipitation studies supported a direct physical interaction between Kv7.4 and Kv7.5 α-subunits in both the A7r5 cell expression system and in the arterial myocytes (Brueggemann et al., 2014b), though it could not be determined whether channels with a particular subunit assembly pattern could account for the distinct responses to the treatments. To control the assembly of α-subunits within tetrameric channels, we constructed concatenated dimers of Kv7.4 and Kv7.5, and concatenated tetramers with alternating Kv7.5 and Kv7.4 α-subunits and measured their behavior in the same smooth muscle cell line.

### Concatenated α-Subunits as a Model to Study Kv Channel Structure/Function

The pore-forming structures of many types of ion channels are thought to form as oligomeric assemblies of α-subunits, with Kv channels forming as tetramers of α-subunits (Catterall, 1988; Brueggemann et al., 2013). The Kv α-subunit proteins are encoded by individual genes in the cell, enabling multiple gene products to assemble in a variety of combinations. In native cell systems that express multiple Kv α-subunit genes, there is a distinct potential for a mix of Kv channel subtypes with different combinations of α-subunits, making the characteristics of tetrameric channels of a particular α-subunit composition difficult to resolve. One experimental approach that has been used to understand how α-subunit assembly affects channel function and pharmacological responsiveness is the overexpression of concatenated DNA constructs that constrain the assembly of α-subunits in certain orders or stoichiometries. For example, Hurst et al. (1992, 1995) created concatenated Kv1.1 constructs, linking four copies of the Kv1.1 cDNA 3′–5′, thereby producing a single DNA vector encoding four Kv1.1 α-subunits, with the C-terminus of one subunit connected to the N-terminus of the next. Expression of this construct in *Xenopus* oocytes yielded Kv currents that resembled the currents generated by overexpression of individual (non-concatenated) Kv1.1 α-subunits, which presumably assemble into tetramers (Hurst et al., 1992).

The characteristics of heteromeric Kv channels were also studied using concatenated constructs in the work of Wimmers et al. (2002). In that study, cDNAs encoding Kv11.1, Kv11.2, and Kv11.3 α-subunits (*erg1*, *erg2*, and *erg3*, respectively) were linked 5′–3′ in pairs, and expressed in Chinese Hamster Ovary (CHO) cells. The authors speculated that expression of alternating α-subunit dimeric constructs (e.g., *erg1*–*erg2*) would result in channels that formed as dimer pairs in a head-to-tail arrangement; for example, *erg1*–*erg2* dimer expression would produce channels with alternating Kv11.1 and Kv11.2 α-subunits in the tetrameric structure (Wimmers et al., 2002). They found that *erg1*–*erg1* dimer expression yielded Kv currents that were similar to currents generated when *erg1* monomer was expressed, with slight differences in kinetics of activation and inactivation; in contrast, *erg1*–*erg2*, *erg1*–*erg3*, and *erg3*–*erg2* dimer constructs generated channels with distinct electrophysiological characteristics, though their regulation by physiological or pharmacological modulators was not examined (Wimmers et al., 2002).

Recent work from the Kurata laboratory utilized concatenated *kcnq2* and *kcnq3* constructs to determine whether Kv7 channel activating drugs exert their effects by binding to individual α-subunits, or by binding to multiple α-subunits within the tetrameric channel structures (Wang et al., 2018; Yau et al., 2018). They utilized mutations that prevent the drug effects to determine that retigabine, a Kv7 channel activator, need interact with only a single retigabine-sensitive α-subunit within a tetrameric Kv7.3 channel to exert its full effect (Wang et al., 2018), whereas another Kv7 channel activator, ICA-069673, required four responsive α-subunits within Kv7.2 channels for its full response (Yau et al., 2018). Although these studies demonstrated that concatenated Kv7 channels are functional in an expression system (*Xenopus* oocytes), the physiological regulation of the channels was not explored.

The findings presented in this article represent the first evidence that heteromeric Kv7.4/Kv7.5 channels can form from expressed concatenated dimers or tetramers of the Kv7.4 and Kv7.5 α-subunits. The features previously found to distinguish Kv7.4/Kv7.5 heteromers from Kv7.4 and Kv7.5 homomeric channels, including their responses to physiological signaling pathways that positively or negatively regulate channel activity, were generally reproduced in M-currents through channels formed from the concatenated Kv7.4 and Kv7.5 α-subunit dimers or tetramers.

In the present study, the concatenated dimer results reveal that the α-subunit order is important, particularly for sensitivity to cAMP/PKA-activating stimuli. M-currents derived from expression of Q4–Q5 dimers were insensitive to forskolin/IBMX, compared with at least modest responsiveness of M-currents derived from expression of Q5–Q4 dimers. This difference may relate to the position of a previously identified N-terminal target for PKA: a serine at position 53 of the Kv7.5 N-terminal segment. In channels formed from individually expressed α-subunits, the Kv7.5 N-terminus would have relatively unconstrained mobility on the cytosolic side of the channel. In contrast, in Q4–Q5 dimers, the Kv7.5 N-terminal S53 site would be found in the concatenated segment between subunits, which may constrain its position in a way that shields it from phosphorylation by PKA. Alternatively, its localization in the segment that tethers two adjacent α-subunits may prevent the phosphorylated serine residue from interacting with the channel structure in a way that would increase its open probability [e.g., by enhancing the channel’s affinity for phosphatidylinositol 4,5-bisphosphate, as previously proposed (Brueggemann et al., 2020)]. According to these lines of reasoning, in the oppositely ordered Q5–Q4 dimer, the position of the Kv7.5 N-terminal S53 site is unconstrained, which would account for its greater sensitivity to cAMP/PKA-activating stimuli. Presumably, the second Q5 in the Q5–Q4–Q5–Q4 tetramer would be “shielded” from PKA regulation, though its enhancement by PKA-activating stimuli was about the same as for Q5–Q4 dimers, suggesting that availability of a single free Q5 N-terminus may be sufficient to confer PKA regulation.

The α-subunit order in concatenated dimers was apparently less important for other features, such as the V_0.5_, suppression by AVP, and the effects of diclofenac on current amplitude and voltage sensitivity, all of which were similar in M-currents of cells expressing either Q4–Q5 or Q5–Q4 dimers. The Q5–Q4–Q5–Q4 concatenated tetramer differed only in its insensitivity to suppression of current amplitude by diclofenac (each of the dimer constructs generated M-currents whose amplitudes at −20 mV were modestly suppressed by diclofenac), though a diclofenac-induced negative shift in V_0.5_ was apparent in both dimeric and tetrameric concatemers. It is notable that these responses to diclofenac are very different from the responses of homomeric Kv7.4 and homomeric Kv7.5. In A7r5 cells expressing Kv7.5 alone, 100 μM diclofenac induced a rapid voltage-dependent block, that was not observed in cells expressing Kv7.4 alone; the effect on Kv7.5 was accompanied by a robust (∼30 mV) negative shift in V_0.5_ (Brueggemann et al., 2011). In the case of Kv7.4 homomers, 100 μM diclofenac enhanced the current amplitude (by ∼40% at −20 mV) and negatively shifted V_0.5_ by ∼10 mV (Brueggemann et al., 2011). Co-expression of Kv7.4 and Kv7.5 in A7r5 cells produced M-currents that were neither enhanced nor inhibited by 100 μM diclofenac at −20 mV, but which demonstrated an intermediate negative shift in V_0.5_ of ∼15 mV (Brueggemann et al., 2011). The native M-currents in freshly dissociated MASMCs behaved like the co-expressed Kv7.4 and Kv7.5 in terms of their insensitivity to diclofenac at −20 mV and a 15 mV negative shift in V_0.5_, which was interpreted as evidence that the native channels are likely Kv7.4/Kv7.5 heteromers (Brueggemann et al., 2011). Like Q5–Q4–Q5–Q4, M-currents in MASMCs were also modestly enhanced by forskolin/IBMX (Mani et al., 2016), and modestly suppressed by AVP treatments (Mackie et al., 2008); these responses are markedly smaller than the responses of Kv7.5 homomers and markedly greater than the responses of Kv7.4 homomers to the same treatments (Brueggemann et al., 2014b; Mani et al., 2016). The similarity of MASMC M-currents to those derived from expression of Q5–Q4–Q5–Q4 concatenated tetramers, and their responses to diclofenac, as well as to forskolin/IBMX and AVP, further suggests that the native myocyte channels may be tetramers of alternating Kv7.4 and Kv7.5 α-subunits in a 2:2 stoichiometry.

While Q5–Q4–Q5–Q4 channels may be functionally present and contribute to M-currents in native myocytes, it cannot be ruled out that other functional channels (e.g., homomers or heteromers of Kv7.4 and Kv7.5) can also contribute to the native myocyte M-currents. In particular, a contribution of homomeric Kv7.4 might explain the findings that the voltage of half-maximal activation (V_0.5_) of concatenated Q5–Q4–Q5–Q4 channels (around −50 mV) is much more negative than that of native MASMC M-currents (−35 mV; Brueggemann et al., 2011). On the other hand, the inhibitory response of the native current to diclofenac (Brueggemann et al., 2011) is lost in concatenated Q5–Q4–Q5–Q4 channels; and the native current in MASMC is markedly inhibited by AVP (100 pM; Mackie et al., 2008), but nearly unaffected in Q5–Q4–Q5–Q4 channels, which might support a contribution of the more responsive homomeric Kv7.5 channels in MASMCs.

### Limitations

There are some limitations of our present study that should be considered. Perhaps the most obvious is the question how well can a concatenated construct mimic the properties of a channel formed from individually expressed α-subunits? We examined several characteristics of the channels (V_0.5_, responses to physiological stimuli, drug responses) and found many similarities to native channels, but there were also some dissimilarities. Concatenation necessarily introduces a link between the C-terminus of one α-subunit and the N-terminus of another, which would not normally be present. Research from our laboratory implicated both the N- and C-termini in the signaling responses of Kv7.5, specifically attributing the response to cAMP/PKA activation to the N-terminal segment and the response to PKC-activating stimuli (including AVP) to the C-terminus (Haick et al., 2017; Brueggemann et al., 2018, 2020). Some differences in signaling sensitivities might therefore arise if the targeted N- or C-terminal segments are tethered to an adjacent α-subunit and less free to interact with kinases or downstream effectors. We did not explore the possibility that longer or more flexible linkers between adjacent α-subunits might result in channels that more closely mimic the regulatory characteristics of channels formed by individual α-subunits.

Signal transduction via Kv7 channels may also involve scaffolding proteins, such as A-kinase anchoring protein (AKAPs; Hoshi et al., 2003; Bal et al., 2010; Zhang et al., 2011) or post-synaptic density protein 95 (PSD-95; Sedó-Cabezón et al., 2015)—it is unclear whether the roles of these scaffolding proteins are altered by concatenation of the α-subunits. Ancillary channel subunits, particularly KCNE proteins, can also modulate the expression and function of Kv channels (Brueggemann et al., 2013); KCNE4 was found to interact with both Kv7.4 and Kv7.5 in MASMCs (Jepps et al., 2015). These interactions, and any changes that result from concatenation of α-subunits, are not considered in the present study. We have detected both AKAP150 and PSD-95, but have not detected expression of any of the five mammalian KCNEs (KCNE1-5) in A7r5 cells (Brueggemann et al., unpublished results).

As summarized in Figure 6A, we consistently observed a difference in voltage dependence of activation of concatenated channel constructs compared with co-expression of the individual α-subunits, with the concatenated channels activating 5–10 mV more negatively. Our previous study implicated the C-termini of Kv7.4 and Kv7.5 as important determinants of the V_0.5_ of the homomeric channels (Brueggemann et al., 2020). Those findings suggested that the cytosolic C-terminal tails of the α-subunits affect the movement of the membrane-resident voltage sensors. Hence, it is not entirely surprising that concatenation of α-subunits, involving links between C-termini and N-termini of adjacent α-subunits, might affect the interactions of C-termini with the voltage sensors, and thereby alter the voltage dependences of the fully formed channels, though we do not have enough structural information to explain why the voltage-dependence is shifted to a more negative range.

Another limitation of our study is that we did not explore an extended range of α-subunit stoichiometries to determine whether other Kv7.4/Kv7.5 subunit configurations are functional e.g., when constrained to assemble as heteromers with 3:1 stoichiometry (Q4–Q4–Q4–Q5, or Q5–Q5–Q5–Q4), or with like subunits adjacent within the tetramer (Q4–Q4–Q5–Q5). Future studies may determine whether these other configurations also reproduce features of the native smooth muscle Kv7.4/Kv7.5 channels. While it is important to emphasize that there may not be a single fixed stoichiometry or arrangement of α-subunits, our study illustrates that Kv7.4 and Kv7.5 heteromerization generates functional channels with distinct properties, and with the ability to serve as downstream targets in receptor-mediated smooth muscle signaling pathways.

### Summary

Heteromeric Kv7.4/Kv7.5 channels formed from concatenated dimers or tetramers of Kv7.4 and Kv7.5 α-subunits conduct M-currents that exhibit many characteristics of native smooth muscle M-currents. Based on these findings, we speculate that in smooth muscle cells, such as MASMCs, where M-currents can be attributed primarily to heteromeric Kv7.4/Kv7.5 channels, the configuration of α-subunits within the tetrameric channel structure is likely alternating Kv7.4 and Kv7.5 in a 2:2 stoichiometry. Though the potential for contributions from channels with other arrangements of α-subunits is not ruled out, the alternating Kv7.4/Kv7.5 α-subunit configuration confers channel properties that are distinct from homomeric channels of either Kv7.4 or Kv7.5 α-subunits, in terms of their sensitivities to signaling pathways that target Kv7 channels to modulate smooth muscle contractility, and in terms of their responses to Kv7 channel modulating drugs. These findings have significance for our understanding of physiological smooth muscle signal transduction, as well as for development of smooth muscle therapeutics that target Kv7 channels.

## Data Availability Statement

The datasets generated for this study are available on request to the corresponding author.

## Author Contributions

LB conducted all the experiments and data analysis. LC designed and constructed the DNA vectors. All authors participated in the research design and writing of the manuscript.

## Conflict of Interest

The authors declare that the research was conducted in the absence of any commercial or financial relationships that could be construed as a potential conflict of interest.
