# Supplementary material for: Heteromeric Channels Formed From Alternating Kv7.4 and Kv7.5 α-Subunits Display Biophysical, Regulatory, and Pharmacological Characteristics of Smooth Muscle M-Currents
Source: Front Physiol. 2020 Aug 12;11:992. doi: 10.3389/fphys.2020.00992 (PMC7434985; doi:10.3389/fphys.2020.00992)
Supplement: Supplementary file 1 [file Data_Sheet_1.pdf]

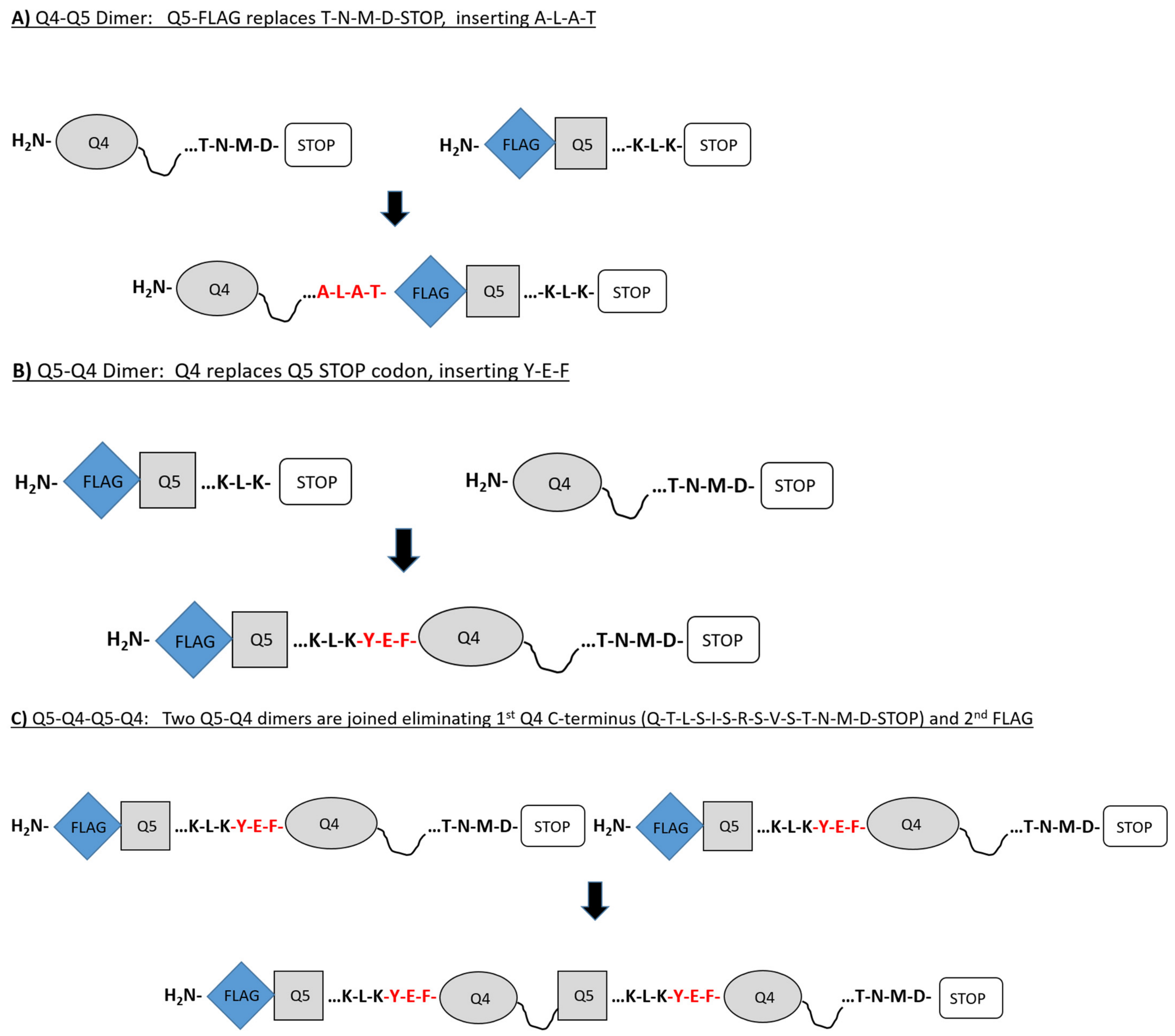

Figure S1. Construction of Concatenated Kv7 Subunits. Schematic representations of a) Q4-Q5; b) Q5-Q4; and c: Q5-Q4-Q5-Q4 constructs. Amino acids inserted as a result of the cloning strategy are shown in red.
